# Surveillance for Emerging Biodiversity Diseases of Wildlife

**DOI:** 10.1371/journal.ppat.1004015

**Published:** 2014-05-29

**Authors:** Laura F. Grogan, Lee Berger, Karrie Rose, Victoria Grillo, Scott D. Cashins, Lee F. Skerratt

**Affiliations:** 1 One Health Research Group, School of Public Health, Tropical Medicine and Rehabilitation Sciences, James Cook University, Townsville, Queensland, Australia; 2 Australian Registry of Wildlife Health, Taronga Conservation Society Australia, Mosman, New South Wales, Australia; 3 Wildlife Health Australia (formerly Australian Wildlife Health Network), Georges Heights, New South Wales, Australia; The Fox Chase Cancer Center, United States of America

Effective surveillance is crucial for early detection and successful mitigation of emerging diseases [1]. The current global approach to surveillance for wildlife diseases affecting biodiversity (“biodiversity diseases”) is still inadequate as demonstrated by the slow characterization and response to the two recent devastating epidemics, chytridiomycosis and white-nose syndrome [2]–[5]. Current surveillance for wildlife disease usually targets diseases that affect humans or livestock, not those impacting wildlife populations. Barriers to effective surveillance for biodiversity diseases include a relative lack of social and political will and the inherent complexity and cost of implementing surveillance for multiple and diverse free-ranging populations. Here we evaluate these challenges and the inadequacies of current surveillance techniques, and we suggest an integrated approach for effective surveillance.

Despite challenges in quantifying the role of disease in species declines [6], there are numerous clear examples of diseases (infectious, toxic, multifactorial, or of undetermined origin) that have caused severe population impacts; for example, avian malaria and poxvirus in Hawaii, diclofenac poisoning in Indian vultures, rinderpest in Africa, bighorn sheep pneumonia, chronic wasting disease, crayfish plague, avian trichomonosis, and Tasmanian devil facial tumor disease [7]–[15].

**Figure 1:**
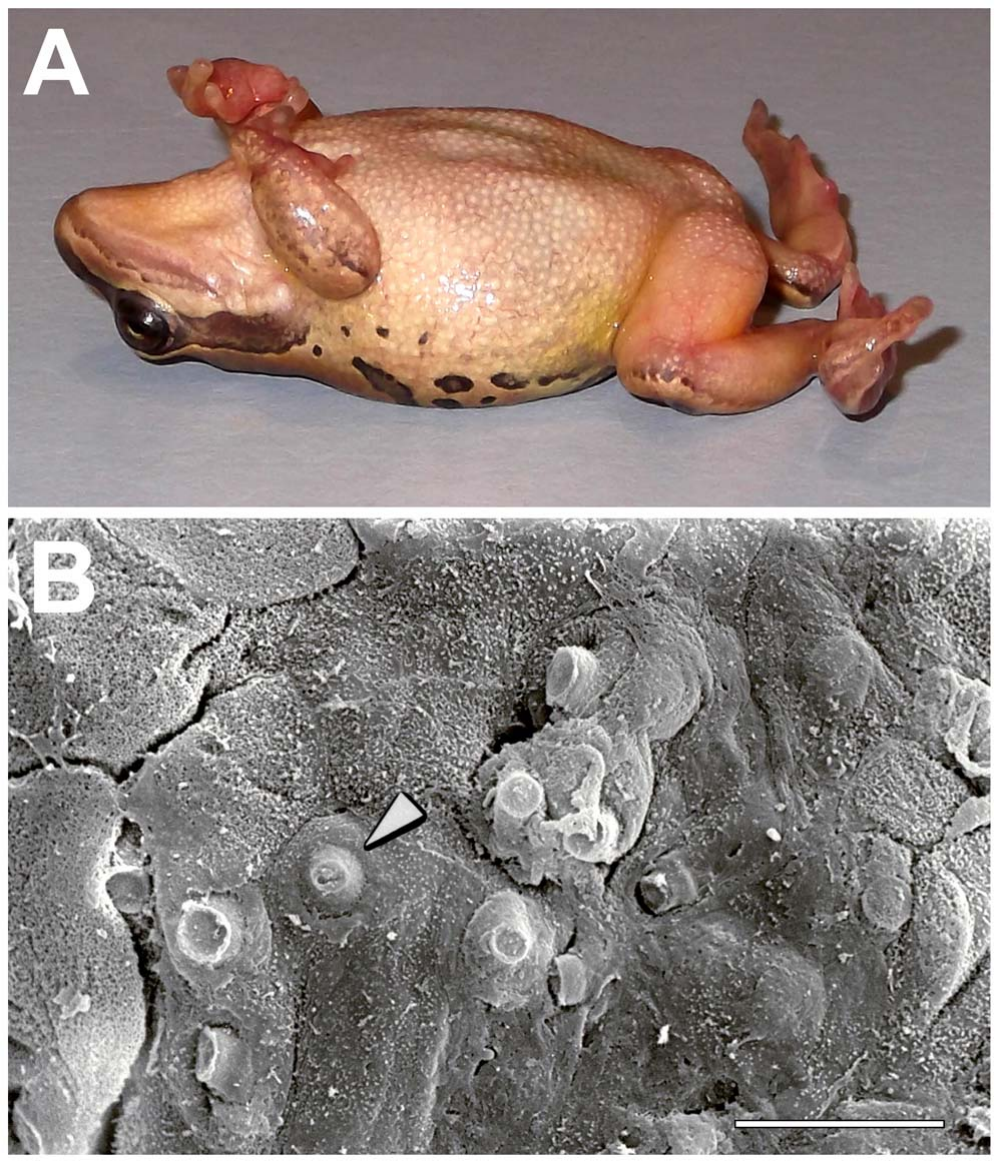
Chytridiomycosis: a catastrophic biodiversity disease causing amphibian declines. Chytridiomycosis emerged in the 1970s but was not detected until the 1990s. (A) An alpine tree frog (*Litoria verreauxii alpina*) with severe chytridiomycosis, showing skin reddening and an inability to maintain normal upright posture; (B) skin surface of a stony creek frog (formerly *Litoria lesueuri*). Many cells are infected with sporangia, pushing discharge tubes (arrow) to the skin surface (scanning electron micrograph). Scale bar = 10 µm.

The emergence of the amphibian fungal skin disease chytridiomycosis is a pertinent example in which a lack of effective disease surveillance contributed to global biodiversity loss (Figure 1) [16]–[18]. Epidemiological investigation did not commence until 15 years after initial declines [19]. Despite recent listing of chytridiomycosis as a notifiable disease by the World Organization for Animal Health (OIE), the extended time before diagnosis very likely contributed to the decline and extinction of at least 200 species of frogs globally, helping to make amphibians the most endangered vertebrate class [3], [20].

Here we define “biodiversity disease” as “a disease that has caused, or is predicted to cause, a decline in a wild species sufficient to worsen its conservation status.” This term can be applied to kingdoms other than Animalia, but those are outside the scope of the current paper. Our aim is to improve wildlife biodiversity disease surveillance, which could have important socioeconomic benefits, including reducing long-term disease management costs, protecting biodiversity and ecosystem services, and contributing to prespillover surveillance for public health and agricultural diseases [21]–[31]. Preventing disease-linked species extirpation will stabilize ecosystems, curtailing cascades of trophic coextinctions and global biodiversity loss [32]–[34]. Biodiversity and ecosystem stability are also increasingly linked with decreased risk of disease emergence [25], [35]–[39].

Current funding priorities for wildlife health surveillance tend to rely on overlap with human and livestock diseases [1]. Cost-benefit analyses applied to zoonotic and agricultural diseases in funding prioritization models, including, for example, the “willingness to pay” framework based on societal values and the concept of paying for “ecosystem services,” typically do not adequately address the consequences of biodiversity loss [4], [40], [41]. Appropriately quantifying the value of biodiversity would assist leveraging more appropriate resource allocation.

Responsibility for wildlife health is often spread across multiple agencies, levels of government, universities, and nongovernment agencies. This fragmentation of accountability may contribute to lower prioritization of biodiversity disease surveillance and control compared with human and livestock health threats, which are managed by specific departments.

To promote effective implementation of surveillance programs, a greater focus on emerging biodiversity diseases is needed in international policy and practice and more support must be given to existing regional wildlife health frameworks, recognizing their crucial role in identifying and managing biodiversity diseases. This recognition should encourage coordination at international, national, and local levels, as well as resourcing on-the-ground surveillance.

Several international bodies concerned with animal health are appropriately situated to take on this coordinating role, and collaborations between bodies such as OIE and the World Conservation Union (IUCN) may provide the necessary transdisciplinary expertise required [3]. The OIE has already taken steps in this direction by listing notifiable and non-notifiable infectious diseases, highlighting current issues through their Working Group on Wildlife Diseases, and developing their “Training Manual on Wildlife Diseases and Surveillance” [42]. International coordination can result in rapid disease assessments, prioritization of resources, and targeted response via regional frameworks for wildlife health (for example, the successfully coordinated, multiagency response to highly pathogenic avian influenza virus, H5N1 [4]).

A number of regional frameworks are already established, while others are new and emerging. With improved funding, regional frameworks for wildlife health will be better equipped to provide direction, facilities, and expertise for surveillance. These centers typically involve collaboration of veterinarians, ecologists, wildlife biologists, microbiologists, and molecular biologists. They require salaries for field staff, epidemiologists, and pathologists; funding for diagnostic testing; and data management systems to collect and analyze surveillance data. Agreement on methodologies, risk assessment pathways, and contingency plans for emerging infectious biodiversity diseases across these regional frameworks will support prompt responses to outbreaks [43].

Current biodiversity disease surveillance is often ad hoc and relies on passive surveillance (data collected from community submissions) or activities that overlap with human and livestock diseases. This approach is unable to elucidate the impact of disease on the population because only the diseased subpopulation is detected, and it is less likely to detect subtle clinical signs or alterations in species fitness, such as reduced fecundity, despite potentially large population impacts [44]–[52]. Some diseases may also be underrepresented due to the cryptic or noncharismatic nature of the hosts, the remote nature of the location, or apathy or acceptance of consequences once a diagnosis has been reached [47], [53]–[55].

Considering the potential deficiencies of current approaches to detect emerging biodiversity diseases, a new, transdisciplinary, *systematic surveillance* approach is needed. Essential elements of this approach are established in many countries, but are not specifically being utilized to detect biodiversity diseases. The following aspects could be incorporated into this approach:

1. **Combine current strategies** (integrate passive and active or general and targeted techniques with outbreak investigations that characterize emerging pathogens or multifactorial disease pathways to enable implementation of effective control) [56]. Surveillance techniques in use for human and domestic animal diseases that may be adapted include:
  - *Disease-specific screening* for incursions of important pathogens.
  - Use of *sentinel species* or individuals at *sentinel locations* (such as key wildlife trade sites) [27], [53], [57]. Species could be ranked for use as sentinels by evaluating:
    - *Species value* based on conservation status, taxonomy, ecosystem representation, and phylogenetic uniqueness.
    - *Sentinel value* based on ecological role (keystone species and predators/scavengers), ease of observation and representative sampling, current level of study, and probability as a disease-emergence host [58].
2. **Target both known and unknown pathogens and hosts and regions predicted to be at high risk for disease emergence through predictive modeling.** Retrospective and risk factor analyses show correlations between the incidence of disease emergence in general and socioeconomic and ecological factors (for example, highly biodiverse developing regions constitute infectious-disease–emergence hotspots which could be targeted [28], [59]–[61]). Deterministic models based on general pathogen characteristics and sensitivity analysis, combined with metagenomic studies, hold potential for predicting future disease emergence [62]–[65].
3. **Ensure spatial and taxonomic representation** to prevent the loss of biodiversity in important taxonomic clades or small regions with high levels of endemism [66].
4. **Focus on multiple biological levels**, such as ecosystems and species [67].
5. **Integrate essential baseline ecological data collection for an understanding of the** ***population impact*** **of disease**. Mark-recapture studies provide long-term data on population dynamics and are appropriate for wildlife population impact assessment, despite imperfect detection [68]. Integration of epidemiological transmission models with disease, population, and environmental data will better elucidate the roles of infectious disease, anthropogenic environmental disturbance, and other factors in driving changes in population structure, distribution, or size [69].
6. **Incorporate** ***self-evaluative*** **mechanisms to ensure adaptability and prioritization strategies**. Strategies should evolve as diagnostic and ecological monitoring techniques emerge, and as global circumstances change [1], [70], [71]. Frameworks for structured decision making and prioritization will ensure that surveillance approaches remain cost effective [72], [73].

In conclusion, we suggest that improved integration, capacity, and a systematic approach to disease surveillance in wildlife are imperative for future biodiversity conservation.
